# Retraction: Age Related Changes in NAD+ Metabolism Oxidative Stress and Sirt1 Activity in Wistar Rats

**DOI:** 10.1371/journal.pone.0313921

**Published:** 2024-11-13

**Authors:** The PLOS ONE Editors

After publication of the Expression of Concern on this article [1, 2], additional concerns were raised about results presented in Figs 7 and 8. Specifically, in Figs 7Biv, 8A and 8C in this article, a number of further blots and/or bands appear similar to each other across two or more figure panels. Some blots and/or bands in these figures of [1] also appear similar to images in Figs 2 and 4 in [3].

The authors have not provided the underlying data that support the published results. As such PLOS cannot resolve the concerns about the similarities between the articles [1, 3, 4] summarized above and in the Expression of Concern [2] and so the *PLOS ONE* Editors retract this article.

All authors either did not respond directly or could not be reached.
